# A randomized comparison of retrograde left‐sided versus anterograde right‐sided ablation of the atrioventricular junction

**DOI:** 10.1002/clc.24038

**Published:** 2023-05-26

**Authors:** Rajdip Dulai, Neil Sulke, Stephen S. Furniss, Anura Malaweera, Pier D. Lambiase, Nikhil Patel, Rick A. Veasey

**Affiliations:** ^1^ Cardiology Research Department Eastbourne District General Hospital, East Sussex Hospitals NHS Trust Saint Leonards‐on‐Sea East Sussex UK; ^2^ Institute of Cardiovascular Science University College of London London UK; ^3^ CVS Healthcare Ltd Eastbourne UK

**Keywords:** atrial fibrillation, atrioventricular node, left‐sided AV node ablation

## Abstract

**Background:**

Catheter ablation of the atrioventricular node (AVN) is an effective treatment for patients with symptomatic atrial fibrillation. This study compares the success rate, procedure time, radiation time, and complication rates of retrograde left‐sided (LSA) and anterograde right‐sided (RSA) AVN ablation in a randomised controlled trial.

**Methods:**

Thirty‐one patients undergoing AVN ablation were randomized to either LSA (15 patients) or RSA (16 patients). Crossover occurred after six unsuccessful radiofrequency (RF) applications.

**Results:**

The LSA cohort had a mean age of 77.00 ± 5.17 and the RSA cohort was 79.44 ± 6.08 (*p* = .0240). There were five crossovers from LSA to RSA and there was one crossover from RSA to LSA. There was no significant difference in ablation time between LSA and RSA (210.40 ± 179.77 vs. 192.19 ± 130.29 seconds, *p* = .748). There was no significant difference in procedure time, fluoroscopy time, radiation dose, or number of RF applications between the two groups. There was 1 (6.67%) serious adverse event in the LSA group and 1 (6.25%) in the RSA group due to femoral hematomas requiring blood transfusion or intervention. There was no significant difference in patient‐reported discomfort between LSA and RSA (16.43 ± 20.67 vs. 17.87 ± 28.08, *p* = .877). The study was stopped before full recruitment due to futility.

**Conclusions:**

Retrograde LSA of the AVN does not reduce RF applications, procedure time, or radiation exposure compared with conventional RSA and cannot be recommended as a first‐line clinical approach.

## Graphical abstract

Retrograde left‐sided atrioventricular node ablation does not reduce radiofrequency ablation time compared with the conventional right‐sided approach and cannot be recommended as a first‐line clinical approach.

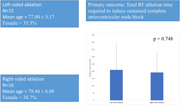

## 1 INTRODUCTION

Atrial fibrillation (AF) is the most common cardiac arrhythmia with 8.5% prevalence in men and 7.1% prevalence in women over the age of 55.^1^ It is estimated that there are 8.8 million adults with AF in the European Union.^1^

Catheter ablation of the atrioventricular node is an accepted and highly effective treatment strategy in patients with AF to improve symptoms and to achieve 100% cardiac resynchronization therapy.^2^ There are two approaches to achieving atrioventricular node block, either the retrograde transaortic approach via the femoral artery approach or the right‐sided approach using the femoral vein.

Right‐sided atrioventricular node ablation is the initial conventional approach; however, up to 18.5% of patients require transferring to a left‐sided approach or otherwise have a challenging procedure with multiple radiofrequency (RF) energy applications and a high radiation exposure.^3^ Previous studies have found that left‐sided ablation is more effective than right‐sided ablation, requiring less applications of RF energy to induce atrioventricular block.^4^, ^5^, ^6^

Performing left‐sided ablation as the initial approach in prospective studies has been shown to have a higher success rate, reduced procedure time, and less radiation exposure than the right‐sided approach.^7^, ^8^, ^9^

However, the right‐ and left‐sided approaches have not previously been directly compared in a randomized trial, raising the possibility of selection bias accounting for the results previously reported. Additionally, there is now potentially a greater clinical requirement to perform left‐sided AV node ablation to avoid displacing or damaging previously inserted pacemaker leads.

This study compares the success of retrograde aortic left‐ and right‐sided approaches to ablation of the atrioventricular node in a randomized controlled trial.

## 2 METHODS

### 2.1 Study design and population

This was a randomized, controlled trial undertaken at East Sussex Healthcare NHS Trust, UK. All patients provided written informed consent. The study was approved by the UK National Research Ethics Service and conducted in compliance with the Declaration of Helsinki.

Subjects were included if their age was greater than or equal to 18 years and if they were referred for atrioventricular node ablation for any appropriate indication. Exclusion criteria included a recent stroke or transient ischemic attack (TIA) within 6 months, myocardial infarction within 6 months, medical conditions limiting expected survival to less than 1 year, moderate to severe aortic stenosis, a history of aortic or mitral valve replacement, and pregnancy or breastfeeding women.

### 2.2 Randomization

Randomization was carried out in a 1:1 ratio (Initial right‐sided or left‐sided ablation) performed using “ralloc,” Stata's randomization process v.16.0. The study‐group assignments were placed in sequential numbered sealed, opaque envelopes, which were opened by a hospital staff member who was not one of the study investigators at the beginning of each procedure.

### 2.3 Preablation protocol

All procedures were performed on uninterrupted anticoagulation with warfarin if the international normalized ratio was less than 2.5. Novel anticoagulants were withheld on the morning of the procedure. All procedures were performed under conscious sedation and local anesthesia.

### 2.4 Retrograde left‐sided ablation protocol

**Figure 1:**
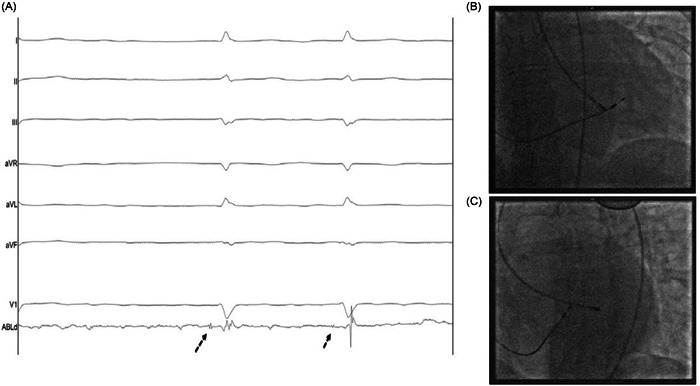
Intracardiac electrogram recordings (A) from the radiofrequency ablation catheter illustrating maximal His potential (arrows) and fluoroscopy location in the posterior–anterior (B) and left anterior oblique (C) views during left‐sided retrograde ablation.

After the infiltration of the local anesthetic, right femoral artery access was achieved using ultrasound guidance. Unfractionated heparin of 100 U/kg was administered after arterial vascular access was achieved. A 4‐mm nonirrigated tip ablation catheter was advanced to the left ventricle via the aortic valve along the anterior septum. RF energy was applied at sites at which the largest possible His bundle deflection was recorded, irrespective of the size of the atrial electrogram (Figure 1). RF applications were delivered for 60–90 seconds at 60 W with a target temperature of 60°C. If atrioventricular block was not achieved after six RF applications, then cross‐over to the right side was permitted. Once the procedure was completed, the ablation catheter and sheath were removed and an angioseal device (Terumo Europe) was used for closure if appropriate.

### 2.5 Right‐sided ablation protocol

**Figure 2:**
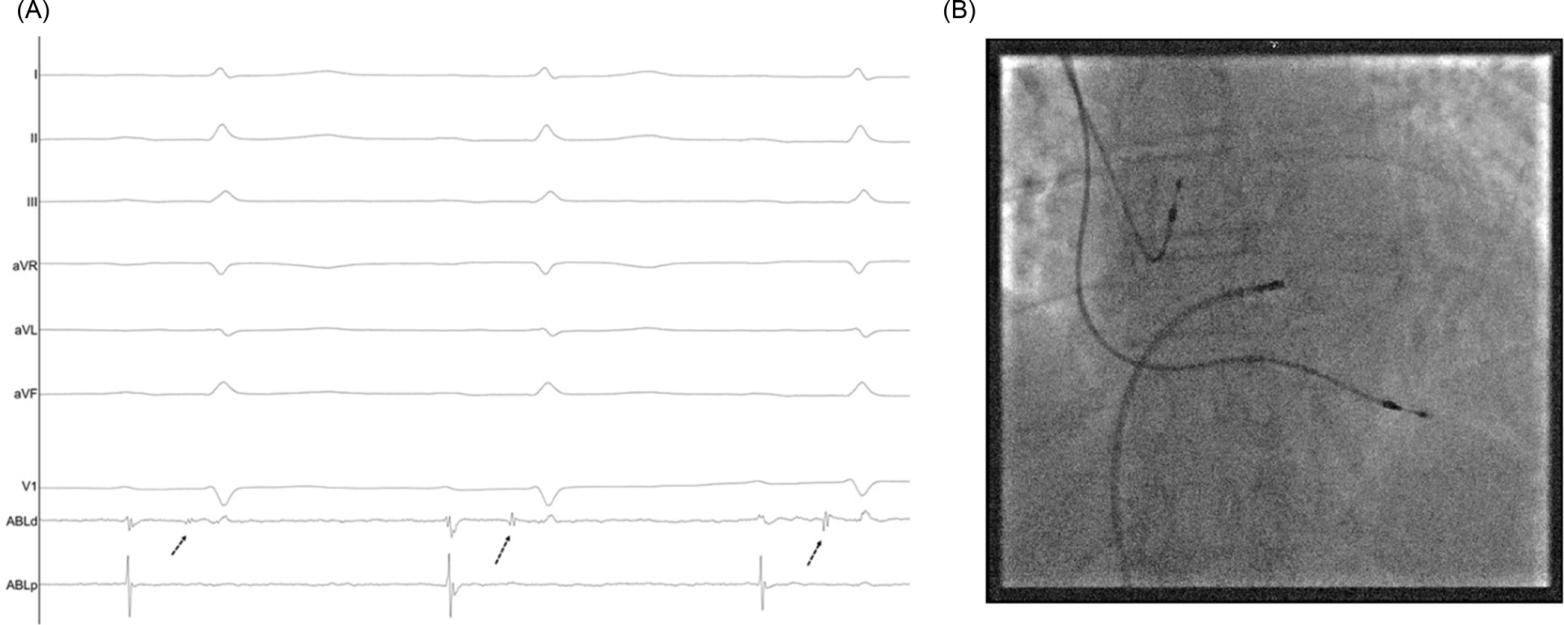
Intracardiac electrogram recordings (A) from the radiofrequency ablation catheter illustrating His potential (arrows) and fluoroscopy location (the mid‐septal region, proximal and inferior to the His bundle) in the posterior–anterior view (B) during right‐sided anterograde ablation.

After the infiltration of the local anesthetic, right femoral vein access was achieved using ultrasound guidance. A 4‐mm nonirrigated tip ablation catheter was advanced to the region of the compact AV node, at the mid‐septal region, proximal and inferior to the His‐bundle recording position (Figure 2). RF energy was applied to these sites. RF applications were delivered for 60–90 seconds at 60 W with a targeted temperature of 60°C. If the atrioventricular block was not achieved after six RF applications, then cross‐over to the left side was permitted. Unfractionated heparin of 100 U/kg was administered if arterial vascular access was required. Once the procedure was completed, the ablation catheter and sheath were removed and manual pressure was applied to achieve hemostasis. If arterial access was also achieved, an angioseal device (Terumo Europe) was used for closure, if appropriate.

### 2.6 Post ablation protocol

Atrioventricular conduction was monitored for 15 minutes after the last RF application. All patients underwent a focussed echocardiogram postprocedure to rule out a pericardial effusion and were monitored according to the local protocol. If there were no adverse events/complications, they were discharged on the same day. Anticoagulation was restarted 6 hours after the procedure if there were no complications. Patients were followed up in the device clinic at 6 weeks.

### 2.7 Outcome measures

The primary outcome measured was the total RF ablation time required to induce sustained complete atrioventricular node block. Secondary outcomes were the comparison of the number of RF applications required to induce complete atrioventricular node block, total procedure time, radiation exposure, rate of the escape rhythm after ablation in beats/minute (bpm), and the number of patients requiring crossover to the alternate technique and adverse events in each group.

Patients were also asked to rate the overall discomfort of the procedure on a scale of 1–100, with 1 being no discomfort and 100 being severe discomfort. Patient discomfort was also assessed using a scale of 1–5, with 1 being no discomfort and 5 being severe discomfort with regard to the following: compression after vascular sheath removal, local anesthesia administration, insertion of vascular sheath and ablation catheter, necessity of immobilization post procedure, limb pain, backache, and bleeding/hematoma post procedure.

### 2.8 Independent data monitoring committee

An independent Data & Safety Monitoring Committee (DSMC) was convened comprising three members, which met to provide independent advice on study conduct, efficacy endpoint, and safety issues. Meetings were held 6 monthly or as required throughout the duration of the trial.

### 2.9 Statistical analysis

The study was powered to address the primary hypothesis that commencing with a left‐sided approach is more effective (RF ablation time) than a right‐sided approach. Souza et al.^5^ reported the time required to induce atrioventricular node block to be 103 and 252 seconds in using left‐ and right‐sided approaches, respectively. Assuming a standard deviation of 225 seconds and 90% power, 96 patients were required for recruitment. The sample size was increased to 100 patients to take into account potential patient withdrawals.

In February 2022, the independent DSMC reviewed and performed an interim analysis after 31 patients had been enrolled. On the basis of these interim results, the sample size was recalculated. Assuming a standard deviation of 155 seconds and 90% power, 3048 patients would have been required to detect a significant difference between the groups. Thus, on the basis of futility analysis, the independent DSMC recommended that enrollment be terminated.

Continuous variables are presented as mean ± SD or median (interquartile range), as appropriate. Categorical variables are presented as absolute numbers and percentages. Continuous variables between groups were analyzed using Student *t*‐test or Mann*–*Whitney *U*‐test*.* Categorical variables between groups were analyzed using the *χ* ^2^ test. An as‐treated analysis of RF ablation time, the number of RF applications and escape rhythm rate outcome was also performed.

The analyses were performed with the use of SPSS software, version 23.0 (SPSS, Inc.).

## 3 RESULTS

### 3.1 Patients and group allocation

Between March 2020 and February 2022, 31 patients were recruited. Of these, 15 patients were randomized to left‐sided ablation and 16 patients were randomized to right‐sided ablation. The clinical characteristics at baseline did not differ significantly between the groups (Tables 1 and 2).

**Table 1:** Baseline characteristics of patients.

	Left‐sided ablation (*N* = 15)	Right‐sided ablation (*N* = 16)	*p* Value
Gender (female), *n* (%)	11 (35.5)	12 (38.7)	.916
Age (years)	77.00 ± 5.17	79.44 ± 6.08	.240
BMI (kg/m^2^)	27.67 ± 4.27	32.23 ± 13.65	.226
CHA2DS2‐VASc score (IQR)	4 (3–5)	4 (3–4)	.902
Comorbidities			
Coronary artery disease, *n* (%)	4 (26.7)	5 (37.5)	.519
Previous myocardial infarction, *n* (%)	1 (6.7)	3 (18.8)	.316
Previous cardiac surgery, *n* (%)	0 (0)	1 (6.3)	.325
COPD, *n* (%)	2 (13.3)	2 (12.5)	.945
Diabetes, *n* (%)	1 (6.7)	2 (12.5)	.583
Thyroid dysfunction, *n* (%)	2 (13.3)	2 (12.5)	.945
Hypertension, *n* (%)	9 (60)	13 (81.3)	.193
Chronic kidney disease, *n* (%)	7 (46.7)	12 (75.0)	.106
Previous stroke, *n* (%)	2 (13.3)	1 (6.3)	.505
Previous TIA, *n* (%)	0 (0)	1 (6.3)	.325
Heart failure, *n* (%)	6 (40.0)	5 (31.3)	.611
Obstructive sleep apnea, *n* (%)	1 (6.7)	2 (12.5)	.583
Atrial fibrillation type			
Paroxysmal, *n* (%)	4 (26.7)	3 (18.8)	.598
Persistent, *n* (%)	2 (13.3)	1 (6.3)	.505
Permanent, *n* (%)	9 (60)	12 (75)	.372
Previous AF ablation			
Cryoablation, *n* (%)	7 (46.7)	5 (31.3)	.379
Radiofrequency ablation, *n* (%)	9 (60)	6 (37.5)	.210
Device type			
Biventricular device, *n* (%)	3 (20)	4 (25.0)	.739
Atrio‐biventricular device, *n* (%)	1 (6.7)	0 (0)	.294
Dual‐chamber pacemaker, *n* (%)	8 (53.3)	5 (31.3)	.213
Single‐chamber pacemaker, *n* (%)	3 (20.0)	7 (43.8)	.157
Indications for ablation			
CRT pacing loss, *n* (%)	1 (6.7)	1 (6.3)	.962
High ventricular rate, *n* (%)	14 (93.3)	15 (93.8)	.962

Abbreviations: BMI, body mass index; COPD, chronic obstructive pulmonary disease; CRT cardiac resynchronization therapy; TIA, transient ischemic attack.

**Table 2:** Previous medication usage.

	Left‐sided ablation (*N* = 15)	Right‐sided ablation (*N* = 16)	*p* Value
Beta‐blockers, *n* (%)	15 (100)	15 (93.8)	.325
Calcium channel blockers, *n* (%)	2 (13.3)	3 (18.8)	.682
Digoxin, *n* (%)	3 (20.0)	4 (25.0)	.739
Flecainide, *n* (%)	3 (20.0)	3 (18.8)	.930
Sotalol, *n* (%)	3 (20.0)	4 (25.0)	.739
Amiodarone, *n* (%)	5 (33.3)	8 (50.0)	.347
Dronedarone, *n* (%)	3 (20.0)	2 (12.5)	.570
Anticoagulation			
NOAC, *n* (%)	13 (86.7)	16 (100)	.131
Warfarin, *n* (%)	2 (13.3)	0 (0)	.131

Abbreviation: NOAC, novel oral anticoagulant.

There were five crossovers from left‐sided ablation to right‐sided ablation. Two out of five crossovers were as per the study protocol with a switch to right‐sided ablation after six unsuccessful RF applications. Three out of five crossovers were performed before any RF application on the left side. This was due to unstable catheter position in two cases, and inability to cross the aortic valve despite repeated attempts in one case. There was one crossover from the right side to left side that occurred as per the study protocol after six failed RF applications.

### 3.2 Outcomes

**Figure 3:**
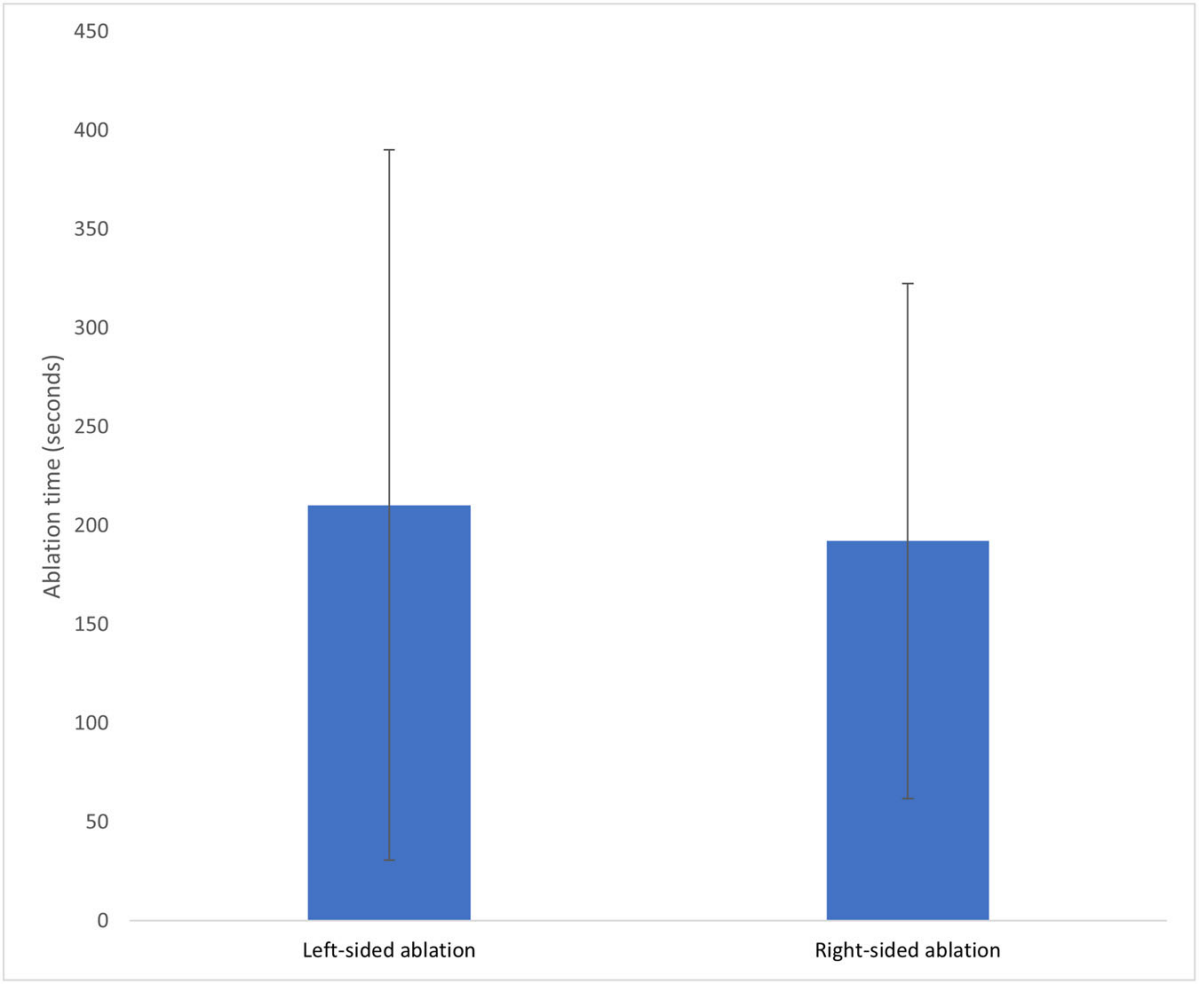
Comparison of mean ablation time ± SD between left‐sided and right‐sided ablation.

There was no significant difference in ablation time between those randomized to left‐sided versus right‐sided ablation (210.40 ± 179.77 vs. 192.19 ± 130.29 seconds, *p* = .748) (Figure 3). There was no significant difference in procedure time, fluoroscopy time, radiation dose, or number of RF applications between the two groups (Table 3). There was no significant difference in the ventricular escape rate between those randomized to left‐sided ablation versus right‐sided ablation (41.7 ± 7.30 vs. 37.7 ± 8.07 bpm, *p* = .197). At 6 weeks, one patient who was randomized to left‐sided ablation had recovery of AV node conduction.

**Table 3:** Comparison of procedural characteristics.

	Left‐sided ablation (*N* = 15)	Right‐sided ablation (*N* = 16)	*p* Value
Procedure time (min)	54.27 ± 11.79	43.69 ± 11.34	.016
Fluoroscopy time (min)	5.95 ± 4.83	2.82 ± 2.55	.031
Radiation dose (cGy cm)	222.47 ± 275.99	104.94 ± 119.95	.131
Number of RF applications (IQR)	1 (1–5)	2.5 (1–4)	.833

Abbreviations: IQR interquartile range; RF, radiofrequency.

### 3.3 Patient‐reported outcomes

**Figure 4:**
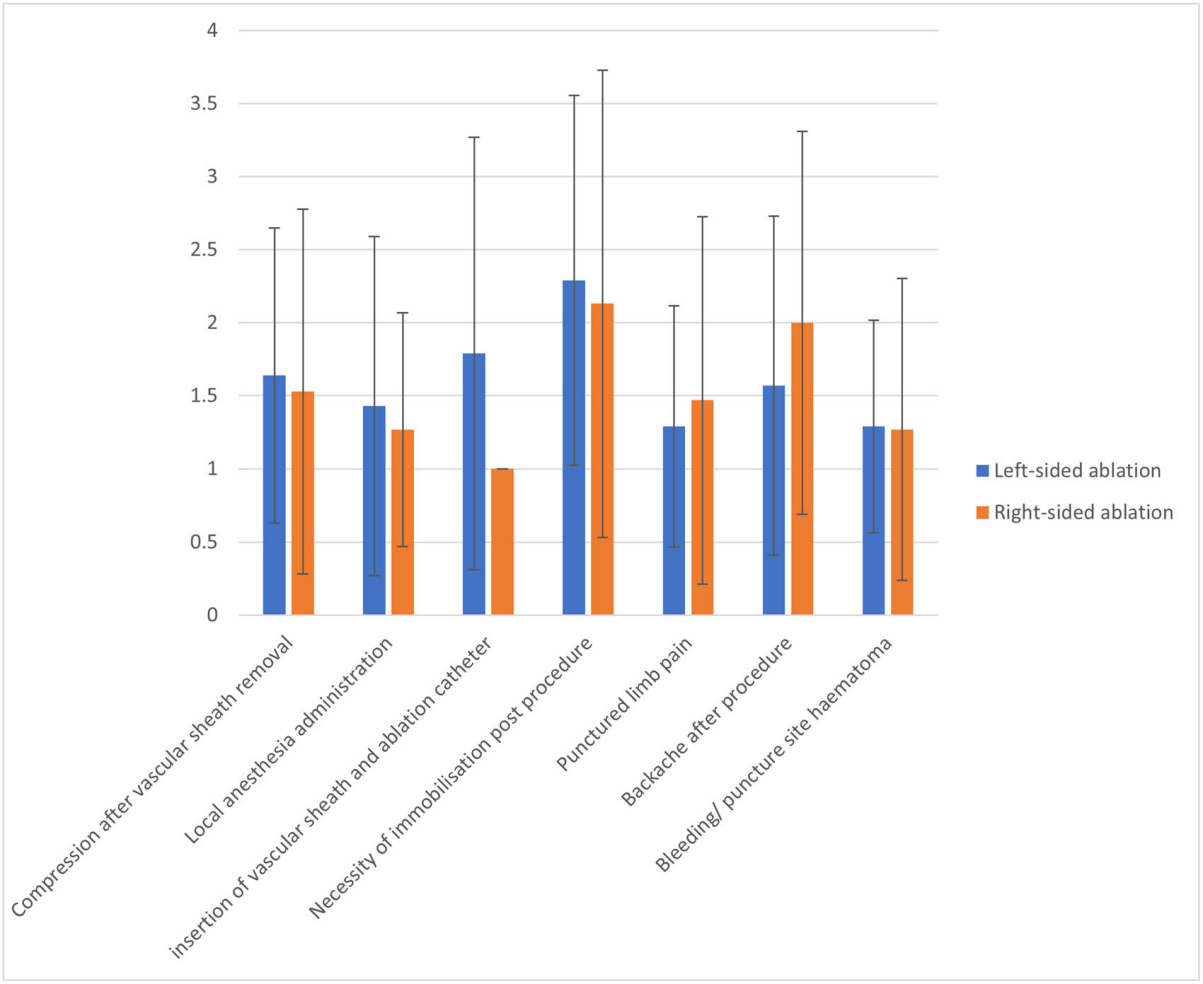
Comparison of patient‐reported comfort (mean score ± SD) between left‐sided and right‐sided ablation.

There was no significant difference in patient‐reported discomfort between left‐sided and right‐sided ablation (16.43 ± 20.67 vs. 17.87 ± 28.08, *p* = .877). There were no significant differences in discomfort with regard to compression after vascular sheath removal (*p* = .798), local anesthesia administration (*p* = .663), insertion of vascular sheath and ablation catheter (*p* = .057), necessity of immobilization postprocedure (*p* = .799), limb pain (*p* = .651), backache (*p* = .360), or bleeding/hematoma postprocedure (*p* = .955) between the two groups (Figure 4).

### 3.4 As‐treated analysis

There was no significant difference in ablation time between those patients undergoing initial RF application on the left‐sided approach versus those patients with an initial right‐sided approach (201.42 ± 177.85 vs. 200.74 ± 141.76 seconds, *p* = .748). There was also no significant difference in the number of RF applications required between left‐sided and right‐sided approaches (1.00 [interquartile range, IQR, 1.00–4.50] vs. 2.00 [IQR, 1.00–4.50]).

There was also no significant difference in the escape rhythm rate between patients treated with a left‐sided approach vs. direct right‐sided approach (41.00 ± 6.78 vs. 38.70 ± 8.58 bpm, *p* = .476).

### 3.5 Adverse and serious adverse events

There was 1 (6.67%) serious adverse event in the left‐sided ablation group and 1 (6.25%) in the right‐sided ablation group. Both serious adverse events were due to hematomas requiring blood transfusion or hospital admission. The serious adverse event in the right‐sided ablation group had crossed over to the left side requiring arterial access. The number of patients with an adverse event was 2 (13.3%) in the left‐sided ablation group. These were due to hematomas not requiring blood transfusion or intervention.

## 4 DISCUSSION

The main finding from this study is that there was no significant difference in the RF ablation time required to induce atrioventricular block between retrograde left and anterograde right‐sided approaches.

Ablation of the atrioventricular node is an effective treatment for symptomatic AF and also to achieve maximal cardiac resynchronization therapy in heart failure patients.^2^ Traditionally this has been performed with a right‐sided approach with success rates of more than 95%. However, in some cases, right‐sided ablation is challenging and leads to procedural failure, with cross‐over to the left‐sided retrograde technique or a repeat procedure.^6^, ^9^, ^10^ In studies performing left‐sided ablation, it has been shown that atrioventricular conduction block can be induced by a lesser number of RF applications resulting in decreased procedure times and fluoroscopy times. Although in many of the studies left‐sided ablation has occurred after attempts at right‐sided ablation, there may have already been a modification of the atrioventricular node before left‐sided ablation was attempted.^5^, ^10^

The proximity of the atrioventricular node to the left‐sided endocardium has been suggested from previous studies in patients who have failed right‐sided ablation. Its proximity to the left‐sided endocardium has been shown in cases in which inadvertent atrioventricular block has been induced while ablating other left‐sided arrhythmias.^7^, ^11^, ^12^ Thus, performing an initial left‐sided atrioventricular ablation has been proposed as an alternative to the right‐sided approach to reduce procedure time, fluoroscopy time, and also reduce the risk associated with pacemaker lead injury or displacement.^9^

Anatomically the common stem of the His bundle is comprised of a nonbranching and branching portion.^13^ The nonbranching portion of the common stem passes through the right fibrous trigone reaching the interventricular septum along the inferior and posterior membranous portion being exposed to the left ventricular myocardium before bifurcating into the right and left bundle branches.^13^, ^14^ In a cadaveric study of 32 human hearts, Massing et al.^14^ showed that the His bundle traversed both the interventricular membranous septum and the left septal crest in 20 cases, and in 4 cases, the His bundle traveled several millimeters below the membranous septum along the left side of the interventricular septum. Right‐sided His bundles were only seen in 5 of the 32 normal human hearts.^14^

Thus, recording His potentials below the aortic valve and between the noncoronary cusp along the membranous septum is achievable. Indeed, in a beagle animal study, Cheng et al.^13^ reported a higher success rate, fewer occurrences of malignant arrhythmias, and less operation and X‐ray time when ablating His bundle potentials from the left‐sided approach when compared to the right‐sided approach.

Souza et al.^5^ conducted a nonrandomized prospective study comparing three groups: right‐sided ablation, failed right‐sided ablation then left‐sided ablation, and initial left‐sided ablation. The number of patients who had direct left‐sided ablation was 7. The number of RF energy applications (3.43 vs. 8.41), fluoroscopy time (10.9 vs. 22.4 minutes), and procedure time (45 vs. 89.1 minutes) was significantly less in the left‐sided ablation group when compared to the right‐sided ablation group.^5^ The fluoroscopy time reported for both groups in the current study is significantly less than that reported by Souza et al.^5^ This may be due to technological advancements and the fact that the procedure is more routine now with operators having greater experience. The operators in this study had completed more than 300 atrioventricular node ablations each before commencement of the study.

In this study, the procedure and fluoroscopy time was significantly greater in those randomized to left‐sided ablation compared to those commencing with a right‐sided approach. This is most likely due to the fact that there was a greater number of crossovers from the left to the right side. Three of the crossovers were performed before any RF was performed due to an unstable catheter position and in one case the operator was unable to cross the aortic valve despite repeated attempts. These procedures had the highest fluoroscopy and procedure times in the entire population studied and thus led to a higher‐than‐expected fluoroscopy and procedure time in the left‐sided group.

More recently, Yorgun et al.^9^ published efficacy and safety outcomes in patients undergoing direct left‐sided ablation. Left‐sided atrioventricular node ablation was successfully performed in 46 out of 47 (98%) patients without any procedural complication. In addition, there was no long‐term recovery of the AV node conduction. Similar to our study, the median number of RF applications for left‐sided ablation was 2 and the mean procedure time was 28.4 ± 2.4 minutes less than that previously reported for right‐sided ablation.^9^ Although the success rate of left‐sided ablation reported by Yorgun et al. was higher than the reported success rate reported in this study, Yorgun et al. only report the outcomes of those undergoing left‐sided ablation and thus there may be selection bias accounting for their results.^9^

One possible cause for the different results seen in this study is that the average age of the patients in the left‐sided cohort was 77.00 ± 5.17 years, which is older than the populations reported by Souza et al.^5^ (56.1 years) and Yorgun et al.^9^ (61.5 years). It is possible that in an older population, there are alterations in anatomy, and consistent contact with the inferior and posterior membranous portion of the left ventricular myocardium is more difficult to achieve due to hypertrophic or fibrotic changes and unfolding of the aorta, which is more common in the elderly and may make catheter manipulation more problematic.^15^

We report a higher complication rate than Yorgun et al.^9^ This may be related to the additional application of a percutaneous collagen closure device in all our patients. There may have been a failure of complete deployment in a few cases, although all operators in our study were very experienced in angioseal deployment. Additionally, Yorgun et al.^9^ stopped novel anticoagulants 24 hours before the procedure, whereas in our study, anticoagulation was continued until the morning of the procedure, increasing the potential for bleeding and hematomas.

In a clinical pathological study of patients who had undergone right and left‐sided atrioventricular ablation, Rizzo et al.^16^ reported the significance of the anatomical variability of the anteroseptal tricuspid valve leaflets commissure in predicting the success of right‐sided and left‐sided ablation. In the patient who had an unsuccessful right‐sided ablation, it was found that the atrioventricular node and His bundle were protected by the continuity between the septal and anterior tricuspid valve leaflets, which was not the case in the patients with successful right‐sided ablation.

In the current study, crossover from right‐sided to left‐sided ablation was permitted after six RF applications. Previous studies have suggested a crossover from the right side to the left side after 3 applications or even up to 10–15 applications.^10^, ^16^ In this study, only one patient required crossover to the left side after six RF applications. Atrioventricular block was achieved after two RF applications on the left side in this patient. Although in most cases atrioventricular block can be achieved in 1–3 RF applications, it is unclear at which stage to use the alternative retrograde left‐sided technique. Operator experience should also be taken into account. It has been found previously that procedural success is related to operator experience.^3^

### 4.1 Study limitations

The main limitation of this study was that the population was elderly with an average age of 77.0 years. Although the age is similar to those reported in registry data, the results of the study may not be applicable to a younger population, although this procedure is less relevant to them as it is rarely used in such younger patients.^3^, ^17^

It has been shown that the rate of cerebral embolization is 58% in patients undergoing left ventricular ablation, as measured by pre‐ and postmagnetic resonance imaging (MRI).^18^ We did not conduct a postprocedure cerebral MRI in our patients; however, to reduce the risk of stroke, interruption of anticoagulation was minimal and all patients undergoing left‐sided ablation were heparinized. Although the risk of subclinical emboli exists when undergoing left‐sided ablation procedures, the clinical significance is uncertain and no clinical cerebral complication occurred in either study cohort.^18^

## 5 CONCLUSIONS

Utilization of an initial retrograde left‐sided approach to atrioventricular node ablation does not reduce RF energy time, applications, or procedure duration compared with the conventional right‐sided venous approach. A direct left‐sided approach is therefore not recommended; however, switching to a left‐sided approach after six unsuccessful right‐sided RF applications is appropriate.

## CONFLICT OF INTEREST STATEMENT

The authors declare no conflict of interest.

## Data Availability

The data that support the findings of this study are available from the corresponding author upon request.
